# A DFT study of structural and electronic properties of copper indium ditelluride Cu_*m*−1_In_*m*_Te_2*m*−2_ with *m* = 2–5 neutral and anion clusters

**DOI:** 10.1039/d5ra03371c

**Published:** 2025-07-01

**Authors:** Kidane Goitom Gerezgiher, Dessie Ashagrie Tafere, Tesfay Gebremikael Teklehaimanot, Ashenafi Belihu Tadesse, Ayalew Manahilie Dinkirie, Hagos Woldeghebriel Zeweldi

**Affiliations:** a Department of Physics, CNCS, Mekelle University P.O. Box 231 Mekelle Ethiopia kidanegoitom5@gmail.com hagos93@mu.edu.et; b Department of Chemistry College of Natural and Computational Science, Mekdela Amba University P.O. Box 32 Tuluawulia Ethiopia; c Department of Chemical Engineering, Bule Hora University P.O. Box 144 Bule Hora Ethiopia; d Department of Electromechanical Engineering, Arba Minch University P.O. Box 21 Arba Minch Ethiopia; e Department of Chemistry College of Natural and Computational Science, Haramaya University P.O. Box 138 Dire Dawa Ethiopia

## Abstract

In this work, the electronic and structural properties of Cu_*m*−1_In_*m*_Te_2*m*−2_ neutral and anion clusters are studied. The simulations are carried out using the QUANTUM ESPRESSO/PWSCF package, based on the density functional theory (DFT) principle, which employs a pseudo-potential with a plane wave basis set. Geometry optimization starting from several initial candidate structures was performed for each cluster size to determine the number of possible minimum-energy isomers for each size. The results show that the lowest-energy structures are cubic, ranging from cluster *m* = 2 to 5, and resemble the chalcopyrite structure. The geometry of neutral and anionic cases exhibits a structural change, including distortion and a transition from two-dimensional to one-dimensional. By considering energetics, *i.e.* HOMO–LUMO gap, binding energy, ionization potential and electron affinity, the relative stability of Cu_*m*−1_In_*m*_Te_2*m*−2_/(Cu_*m*−1_In_*m*_Te_2*m*−2_)^−^ was measured. From the most stable energy structures, CuIn_2_Te_2_/(CuIn_2_Te_2_)^−^ were found to have enhanced chemical stability relative to their neighbours. They are a magic-number species. The binding energy and HOMO–LUMO gap of CuIn_2_Te_2_/(CuIn_2_Te_2_)^−^ clusters show the most significant value, which indicates high chemical stability. The adiabatic ionization potential of the cluster decreases monotonically, showing favor for metallic character as cluster size increases. Both clusters' vertical/adiabatic detachment energies also show a slight odd–even oscillation with an increasing tendency as a function of cluster size. This indicates that the successive increase in metallic atoms results in a decrease in nonmetallic favor. We also analyse the partial charge density of the optimized geometries for both anion and neutral clusters. The numerical value indicates that these clusters, including photovoltaic solar cells and other devices, make a significant contribution to semiconductor design.

## Abstract 2

In this work, the electronic and structural properties of Cu_*m*−1_In_*m*_Te_2*m*−2_ neutral and anion clusters are studied.

## Graphical abstract

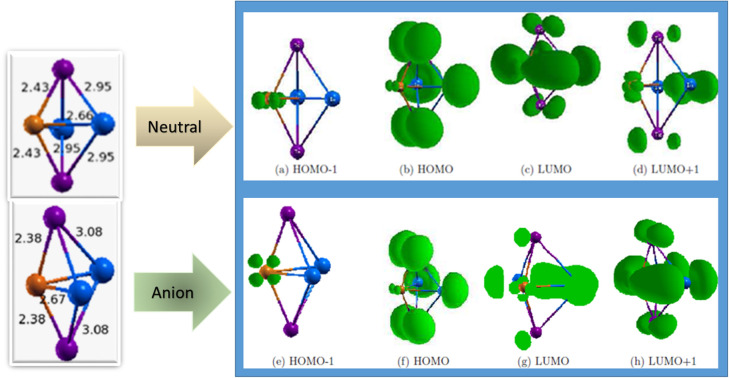

## 1 Introduction

Chalcopyrite semiconductor nanoclusters composed of elements from groups I, III, and VI have garnered significant attention in nanotechnology due to their unique properties and potential applications.^1^ These materials exhibit tuneable optical and electronic characteristics, making them suitable for various applications,^2,3^ including photovoltaics and energy storage devices. Moreover, transition metal clusters exhibit properties that significantly differ from those of individual atoms, molecules, or bulk materials, primarily due to their unique size, shape, and electronic structure.^4^ These clusters, often characterized by quantum confinement effects, demonstrate distinct optical, electronic, and catalytic properties not observed in larger-scale materials.^5^ Transition metal clusters can exhibit superatom behaviour, where their electronic properties resemble those of simple atoms, particularly in their optical and magnetic spectra.^6^

Integrating coinage metals with semiconductors to form ternary chalcopyrite (I–III–VI_2_) is crucial in developing low-cost thin-film solar cells. These materials, particularly copper and silver-based chalcogenides, exhibit promising optoelectronic properties, making them suitable for photovoltaic applications.^7,8^ Materials like copper indium diselenide (CuInSe_2_) have an optical band gap of 1.04 eV,^9^ while Cu_2_SnS_3_ has a band gap of 1.23 eV,^10^ both of which are ideal for solar absorption. The investigation of the structural and electronic properties of pure copper, gold, silver, and zinc clusters, as well as semiconductors, reveals significant insights into their behaviour and interactions. To date, various studies utilizing density functional theory (DFT) have highlighted the unique characteristics of these materials, particularly in terms of stability, electronic structure,^11^ and potential applications in optoelectronics. It should be noted that CuInTe_2_ (CIT) can indeed be produced for thin-film photovoltaic solar cells using methods such as electrodeposition and pyrolysis.^12^ The electrochemical synthesis of CIT has shown promising results, particularly in terms of material properties and efficiency.

Various materials are found to be suitable for solar energy conversion through the photovoltaic method. Still, most of the interest is concentrated on ternary chalcopyrite I–III–VI semiconductors, which can be fabricated into thin-film solar cells at low cost using the electrochemical method.

## 2 Computational details

To study the structural and electronic properties of Cu_*m*−1_In_*m*_Te_2*m*−2_ neutral and anion clusters. QUANTUM ESPRESSO/PWSCF package is applied. The package employs density functional theory (DFT) using a pseudopotential and a plane-wave basis set. The input in the ESPRESSO/PWSCF package is the configuration of atoms encapsulated in a supercell that is repeated periodically. The simplest output is the electronic structure and energy of the atom configuration, as well as the atom's movement. The electronic structure is a spatial array of electronic density and an electronic energy level population that could be integrated to give the state's electronic density.

All calculations will be performed using density functional theory (DFT) within the ultra-soft pseudopotential plane-wave method. During structure optimization, we have used a cubic super cell of edge size 20 Å and periodic boundary conditions are imposed. Besides, only the (Γ) point is used to sample the Brillion zone (the Brillion zone is sampled by a single *k*-point (Γ)) due to the large size of the super cell. Gaussian smearing of 0.01 eV was used to perform the states' density. For each system, the cut-off energy for the plane wave was set to be 240 eV.

The structures were considered converged when the force on each ion was less than 0.0001 eV eV^−1^, using a Sham energy functional. The conjugate gradient method was employed, allowing for the quenching of various planar and three-dimensional configurations. The number of electrons considered in each species with their valence electron configuration is 3d^10^4s^1^ for Cu, 5s^2^5p^1^ for In, and 5s^2^5p^4^ for Te. In all cases, we have spanned as much geometry as possible, both with and without symmetry considerations, and by interchanging copper, indium, and tellurium atoms to ensure that the possible lowest energy geometry has been found.

Two global optimization techniques were employed, namely the SK and ABC algorithms, which allowed access to a broader spectrum of pre-selected structural candidates.^11^ The effectiveness and reliability of these search methods have been well established in numerous published studies. To search for the lowest energy structures of clusters, twenty to thirty initial configurations were considered. The stable neutral clusters' equilibrium geometries are considered a starting point for the geometry optimization of the ionized aggregates.

## 3 Results and discussion

The so called ternary solid semiconducting materials that contain three or more chemical elements are belonging to group I–III–VI of the periodic table. They usually involve two metals and one chalcogen. The materials CuInX_2_ (X = S, Se, Te) are significant in optoelectronic applications due to their direct band gap of approximately 1.5 eV, which is ideal for solar cell technologies. CuIn(SeTe)_2_ can achieve band gaps below 1 eV, which is advantageous for tandem solar cell applications, allowing for better current matching.^13,14^ These materials exhibit unique properties that enhance their performance in various applications, particularly in photovoltaic devices. This gap enables them to be an efficient absorber of sunlight and thus potential photovoltaic solar cells.^15^ The elasticity and thermodynamic properties of CuInX_2_ materials are crucial for their stability under varying temperature and pressure conditions, which are also essential for their practical applications.^16^ These semiconducting materials are a unique class of materials that are intermediate between molecular clusters and solids. They have been extensively studied at the nano level due to their unique size-dependent electronic and optical properties.

In solid crystals, electronic energy levels form quasi-continuous bands, while small clusters exhibit discrete energy levels. As cluster size decreases near the exciton Bohr radius, the bulk semiconductors' continuous band structure transitions to discrete, atomic-like states. Conversely, as cluster size increases, these discrete levels converge into a pseudo-continuum resembling the bulk solid-state band structure. The HOMO–LUMO (H–L) gap widens with decreasing size, particularly in metals, due to quantum size effects, significantly altering electrical, optical, and magnetic properties. While size-dependent tuning offers potential, challenges arise in applications such as nanoelectronics, superlattices, and biological labelling when dealing with very small particles. Recent advances in transition metal research have enabled property tuning through stoichiometric control in mixed ternary nanocrystals. The periodicity of the crystal lattice enables the application of Bloch's theorem, leading to the formation of energy bands where electrons can occupy a range of energy levels.^17,18^

Ternary chalcopyrite clusters have been extensively studied due to their significance in material chemistry and physics, as well as their potential applications in electronics.^19,20^ These semiconducting clusters typically crystallize in a cubic diamond-like structure similar to zinc blende compounds, where each atom occupies the centre of a tetrahedron formed by atoms of a different kind. Chalcopyrite compounds, particularly Ga/Al/Eu-doped indium telluride, are integral to various technological applications^21^ due to their unique properties. These materials exhibit significant potential in fields such as photovoltaics, thermoelectrics, and optoelectronics, making them valuable for advancing energy conversion technologies. This section intends to investigate stoichiometric Cu_*m*−1_In_*m*_Te_2*m*−2_ (*m* = 2–5) clusters using the DFT method.

The stable geometric structures of Cu_*m*−1_In_*m*_Te_2*m*−2_ neutral and anion clusters were addressed using *ab initio* calculations based on density functional theory. In searching for the three least energy structures arranged in ascending order of their energies, a lot of possible initial structures which include 1, 2 and 3 dimensions, were considered. During the optimization process, binding energy, HOMO–LUMO gap, dissociation energy, second-order energy difference and ionization potential are calculated. The binding energy is defined as the amount of energy required for its creation or the amount of energy needed to be added to the system to break it apart. The binding energy formed by pair (A_*x*_B_*y*_) can be calculated as:1
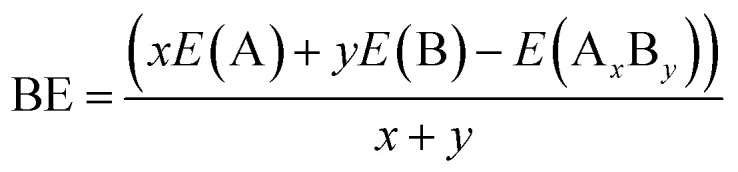


The binding energy of a certain cluster formed by three different atoms A, B and C is defined as:2
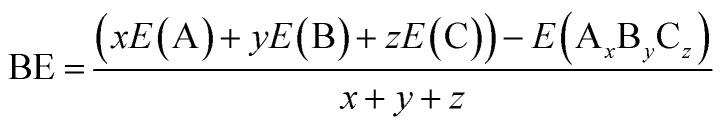
where *x*, *y* and *z* will be the number of individual atoms, *E*(A), *E*(B) and *E*(C) are the minimum energy of the isolated atoms A, B, and C respectively, and *E*(A_*x*_B_*y*_C_*z*_) is the minimum energy of respective clusters.

The BE of Cu_*m*−1_In_*m*_Te_2*m*−2_ neutral and anion clusters are calculated using eqn (2), and their values are tabulated.

The energy difference between the highest occupied molecular orbital (HOMO) and lowest unoccupied molecular orbital (LUMO) reflects the ability for electrons to jump from occupied orbital to unoccupied orbital. It represents the ability of the molecular orbital to participate in chemical reactions to some extent.^11,22–24^ A larger HOMO–LUMO gap corresponds to weaker chemical reactivity, indicating greater electronic stability. The energy difference between the HOMO and LUMO would be calculated using:

3 [HOMO–LUMO]_gap_ = *E*_LUMO_ − *E*_HOMO_

Another point of deal to verify the stability of clusters is so called ionization potentials (IP) and electron affinities (sometimes referred to as electron detachment energy *i.e.* −EA or DE). They may be determined for two cases, vertical and adiabatic. The vertical ionization potential (VIP) is defined as the difference in energy between the lowest energy structure of the neutral and the cation with the geometry of the neutral. In contrast, the vertical detachment energy (VDE) is the difference in energy between the lowest energy of the anion and the neutral with the anion's geometry.^25–27^ This differs in that the adiabatic case is simply the difference in energy between the lowest energy structures for both the neutral and cation or neutral and anion for the AIP and ADE, respectively. The adiabatic ionization energy is the energy required to form a molecular or atomic cation in its ground state *via* the loss of an electron from the ground state of the neutral system in the gas phase.^28,29^ The electron affinity (EA) is defined similarly to the adiabatic ionization energy, and the vertical electron attachment energy is similar to the vertical ionization energy, but the quantities correspond to the enthalpy changes at 0 K. Each of these quantities can be represented mathematically, *via*,^30,31^

4 AIP = *E*_ground state_^+^ − *E*^0^_ground state_

5 VDE = *E*^0^_geometry of anion_ − *E*_ground state_^−^

6 ADE = *E*^0^_ground state_ − *E*_ground state_^−^

where *E* is the energy, and the superscripts represent the charge of the cluster.

These values hold high importance. For example, if a cluster has low electron detachment energy, the neutral cluster prefers not to accept one unit of charge; however large electron detachment energy is an indication that the anion is the more stable species. If a cluster has a large ionization potential, it is indicative of the stable nature of the neutral cluster *versus* the cation. The preceding section is about examining the growth pattern of these clusters as a function of the number of Cu_*m*−1_In_*m*_Te_2*m*−2_ (*m* = 2–5), molecular units.

### 3.1. Lowest energy geometries of Cu_*m*−1_In_*m*_Te_2*m*−2_ for *m* = 2–5 neutral and anion clusters

#### 3.1.1. CuInTe_2_/(CuInTe_2_)^−^ clusters

**Fig. 1:**
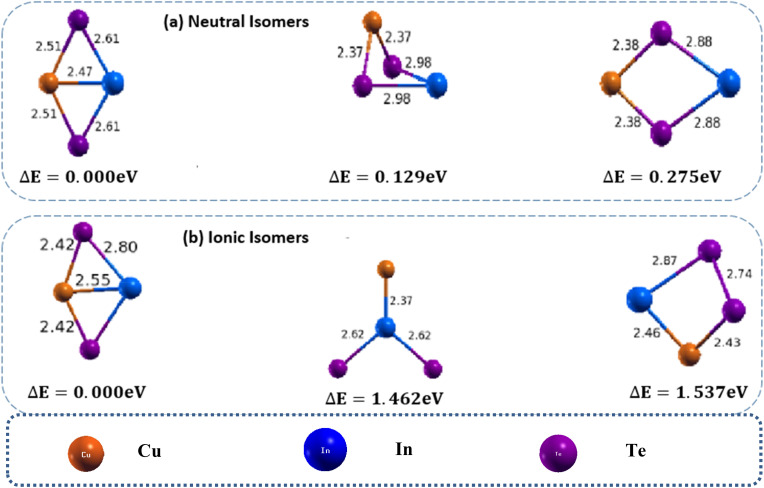
Optimized geometry of neutral CuInTe_2_ (a) and anion (CuInTe_2_)^−^ (b) clusters with corresponding relative energy Δ*E*, with respect to the lowest energy structure. Clusters with higher energy are labelled as (b) and (c) in increasing order. The total energy is normalized to the energy of the lowest energy cluster.

Our calculations for these clusters revealed three stable and distinct structures for the tetraatomic neutral cluster of CuInTe_2_: one 3D and two 2D (Fig. 1a–c). The 2D cluster features a Cu bond crossing in the middle, with bond lengths of Cu–In, Cu, and In–Te approximately 2.47 Å, 2.51 Å, and 2.61 Å, respectively, which is found to be the most stable structure. This structure, named isomer 1(a), is in the form of a planar rhombus and has an energy 0.129 eV lower than that of 1(b) and 0.275 eV lower than that of 1(c), respectively. It has a bond angle of 63.3° between Cu, In, and Te. Isomer 1(b) is a distorted di-bridge structure, and isomer 1(c) is a planar di-bridge structure. The binding energy and H–L gap energy of isomer 1(a) are found to be 2.942 eV and 0.577 eV, respectively. This clearly shows that the least energy isomer of CuInTe_2_ adopts a planar rhombus structure because it offers an optimal configuration for electronic stabilization, coordination environment, and steric minimization, resulting in the observed binding energy and H–L gap.

In the anion counterpart, the low-lying isomers are two-dimensional structures, as shown in Fig. 1. The atoms prefer the distorted structure and the lowest energy isomer compared to the neutral isomer. The bond length of the anion case is also different from that of the neutral isomers. The bond length of Cu–In, Cu, and In–Te in the first isomer, Fig. 1(a), are 2.55 Å, 2.42 Å and 2.79 Å, respectively, which are totally different from the neutral isomer. Isomer (1b) is changed from neutral three-dimensional to two-dimensional with a bond length of Cu–In and In–Te of 2.37 Å and 2.62 Å, respectively. Isomer 1(c) is a distorted square having a bond length of Cu–In and Cu–Te 2.46 Å and 2.43 Å, respectively. The increase in bond length for the anion CuInTe_2_ compared to the neutral molecule is a consequence of altered electronic structure, weakened bonding interactions, and structural adjustments in response to the ionization process. The ADEs/VDEs values for all e isomers of (CuInTe_2_) are calculated as 3.53/3.78, 4.12/2.58 and 2.26/2.482.48 eV the planar rhombus, distorted di-bridge and planar di-bridge structures, respectively. The planar rhombus is the most favourable isomer in terms of overall stability and adaptability after electron detachment, making it a likely candidate for the dominant structure in experiments or applications.

##### 3.1.1.1. CuIn_2_Te_2_/(CuIn_2_Te_2_)^−^ clusters

**Fig. 2:**
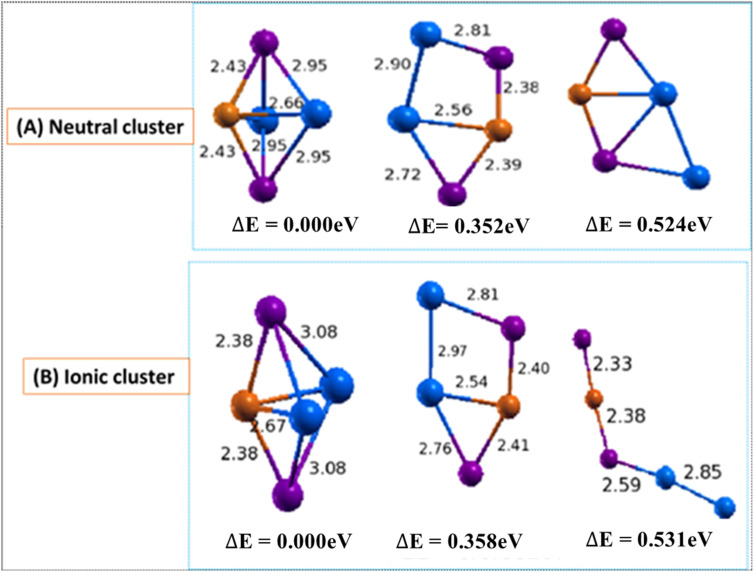
Optimized geometry of neutral Cu_*m*−1_In_*m*_Te_2*m*−2_ (A) and anion (Cu_*m*−1_In_*m*_Te_2*m*−2_)^−^ (B) (*m* = 2) clusters with corresponding relative energy Δ*E*, with respect to the lowest energy structure. Clusters with higher energy are labelled as (b) and (c) in increasing order. The number in front signifies the size of the *m* unit, and the total energy is normalized to the energy of the lowest energy cluster.

The low lying isomers of Cu(In)_2_Te_2_/(Cu(In)_2Te2_)^−^ clusters are shown Fig. 2. The first two lowest energy structures of this neutral cluster are 3D with trigonal bi-pyramidal and distorted trapezoid structures respectively. Isomer 2(b) and isomer 2(c) are 0.352 eV and 0.524 eV higher than isomer 3(a) respectively. The calculated bond length of Cu–Te, Cu–In and In–Te are found to be 2.43 Å, 2.67 Å and 2.95 Å respectively. The binding energy and the HOMO–LUMO gap are calculated and their values are 3.018 eV and 2.072 eV respectively. The calculated values of ADEs/VDEs for each isomer are 3.69/3.83, 3.84/3.99, and 3.88/6.12 eV respectively, in order of increasing energy. The significant difference in the VDE of isomer 3 suggests it might play a dominant role in experimental observations, especially in processes sensitive to electron binding or structural reorganization. The difference between VDE and ADE indicates the degree of structural relaxation after electron detachment. A large difference suggests significant structural reorganization after electron removal, indicating a flexible or adaptable geometry.

In the anion part, the average bond length of Cu–In, Cu–Te and In–Te of isomer 2(a), are 2.67 Å, 2.38 Å and 3.08 Å, respectively. Isomers 2(b) and 2(c) of the anion are quite different in geometry from the neutral cluster. Considering isomer 2(a), the bond length of Cu–Te less than isomer 2(a) of neutral cluster. But the bond length of In–Te is higher than isomer 2(a) of neutral one. Isomer 2(c) is linear by breaking its bonds. The binding energy and HOMO–LUMO gap of (CuIn_2_Te_2_)^−^ are calculated to be 1.178 eV and 2.157 eV respectively.

##### 3.1.1.2. Cu_2_In_3_Te_4_/(Cu_2_In_3_Te_4_)^−^ clusters

**Fig. 3:**
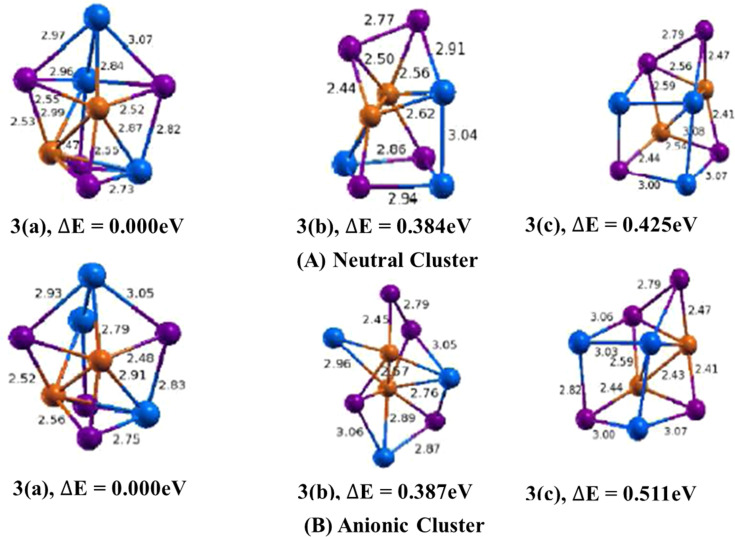
Optimized geometry of neutral Cu_*m*−1_In_*m*_Te_2*m*−2_ (A) and anion (Cu_*m*−1_In_*m*_Te_2*m*−2_)^−^ (B) (*m* = 2) clusters with corresponding relative energy Δ*E*, with respect to the lowest energy structure. Clusters with higher energy are labelled as (b) and (c) in increasing order. Number in front signifies size of *m* unit and total energy is normalized to the energy of the lowest energy cluster.

The lowest energy geometry of Cu_2_In_3_Te_4_ neutral cluster shown in (Fig. 3, 3(a)) is a distorted cube capped with an indium atom having a di-bridge planar rhombus structure of two copper and two tellurium atoms. Isomer 3(b) is a square planar bonded with the square pyramid. Similarly, isomer 3(c) features a distorted cube with an emerging tellurium atom at its apex. The lowest energy structure is 0.384 eV lower in energy than the second and 0.425 eV lower than the third isomers. In isomer 3(a), the average bond lengths are calculated to be 2.90 Å, 2.54 Å, and 2.91 Å for Cu–In, Cu–Te, and In–Te dimmers, respectively. It also features a Cu–Cu bond with a 2.47 Å bond length and an In–In bond with a 3.14 Å bond length. The binding energy per atom and the HOMO–LUMO gap are 3.3.018 eV and 0.699 eV respectively.

In the anion case, the geometry isomer 3(a) loses one In–Te bonding. This clearly shows that when Cu_2_In_3_Te_4_ neutral becomes an anion, the bonding energy between In–Te is lost and becomes unstable. As shown in Fig. 3, 3(a), the bond length of Cu–In and Cu–Te is different from neutral and ranges from 2.79–2.91 Å and 2.48–2.56 Å, respectively. The calculated values of ADEs/VDEs for each isomer are 3.76/3.92, 3.45/3.74, and 3.70/4.28 eV, respectively, in order of increasing energy. The minimal relaxation (0.160 eV), indicating a rigid geometry. Thus, isomer 1 is the most stable and likely the dominant form in a relaxed equilibrium scenario due to its highest ADE and rigid structure.

##### 3.1.1.3. Cu_3_In_4_Te_6_/(Cu_2_In_3_Te_4_)^−^ clusters

**Fig. 4:**
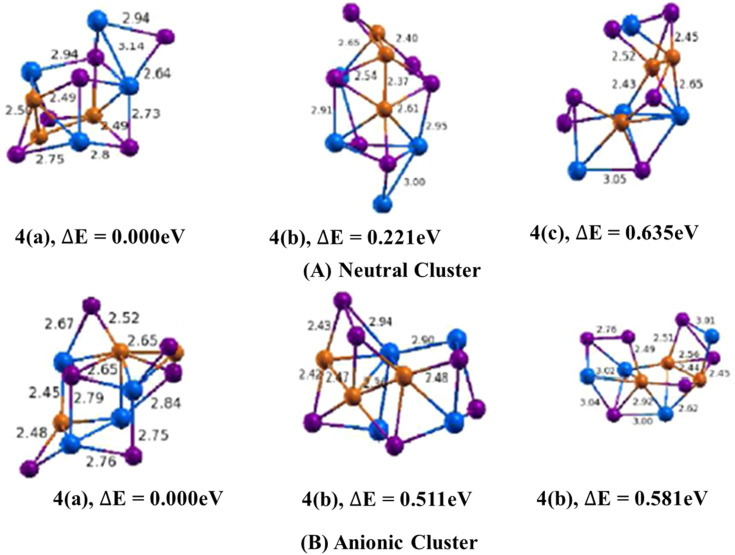
Optimized geometry of Cu_*m*−1_In_*m*_Te_2*m*−2_ (A) and (Cu_*m*−1_In_*m*_Te_2*m*−2_)^−^ (B) (*m* = 4) clusters with corresponding relative energy Δ*E*, with respect to the lowest energy structure. Clusters with higher energy are labelled as (b) and (c) in increasing order. The number in front signifies the size of the *m* unit, and the total energy is normalised to the energy of the lowest energy cluster.

The lowest energy geometry of Cu_3_In_4_Te_6_ neutral cluster shown in (Fig. 4, 4(a)) is a three-dimensional structure with a distorted pentagonal structure having an extension of a di-bridge planar rhombus. The average bond angle between Cu–Te–Cu is found to be 62.6^°^, with an average bond length of Cu–Te and In–Te of 2.50 Å and 2.80 Å, respectively. The H–L gap energy of this structure is found to be 0.524 eV, and the binding energy is 3.503 eV. The calculated values of ADEs/VDEs for each isomer are 3.68/4.58 eV, 3.77/3.37 eV and 3.48/3.65 eV respectively, in order of increasing energy.

In the anion counterpart, isomer 4(a) is shown in (Fig. 4) having a geometry which is a distorted cube with a preference surface site of tellurium atoms and an interior site preference of copper atom. The bond lengths of Cu–Cu, Cu–In, Cu–Te and In–Te are tabulated. The values are calculated to be 2.65 Å, 2.48 Å, 2.55 Å and 2.74 Å, respectively. The lowest energy isomer 4(a) is 0.511 eV lower than the second and 0.581 eV than the third isomer. The binding energies and HOMO–LUMO gap are 0.969 eV and 1.298 eV, respectively.

##### 3.1.1.4. Cu_4_In_5_Te_8_/(Cu_4_In_5_Te_8_)^−^ clusters

**Fig. 5:**
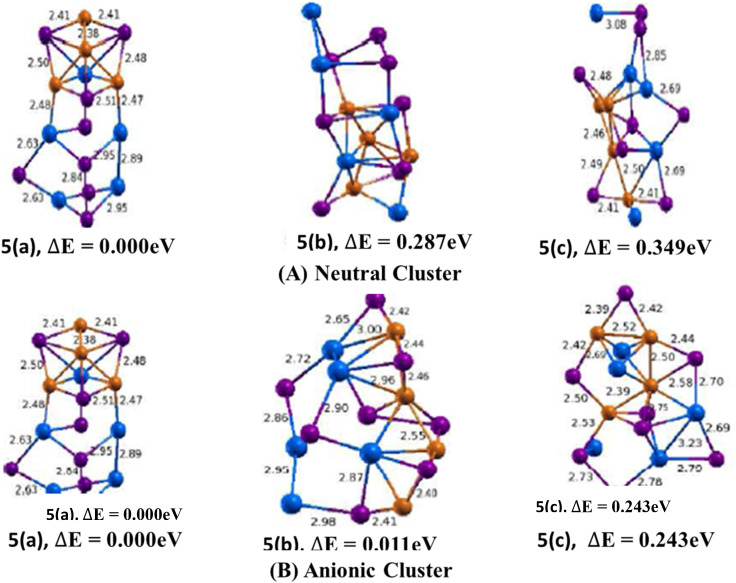
Optimized geometry of neutral Cu_*m*−1_In_*m*_Te_2*m*−2_ (A) and anion (Cu_*m*−1_In_*m*_Te_2*m*−2_)^−^ (B) (*m* = 5) clusters with corresponding relative energy Δ*E*, with respect to the lowest energy structure. Clusters with higher energy are labelled as (b) and (c) in increasing order. Number in front signifies size of *m* unit and total energy is normalized to the energy of the lowest energy cluster.

For neutral cluster, three isomers are considered. The lowest energy structure of Cu_4_In_5_Te_8_ cluster shown in (Fig. 5, 5(a)) resembles to 3D growth of the cubical tetragonal structure which is commonly called chalcopyrite. The calculated bond length of Cu–In, Cu–Te and In–Te are found to be 2.81 Å, 2.64 Å and 2.38 Å respectively. It also has Cu–Cu and In–Te bonds with average bond lengths of 2.48 Å and 2.83 Å. The binding energy per atom and H–L gap are calculated to be 3.527 eV and 1.007 eV, respectively. The lowest energy structure is 0.287 eV lower in energy than the second and 0.349 eV lower than the third isomers. The calculated values of ADEs/VDEs for each isomer are 2.33/2.43 eV, 2.27/2.49 eV, and 2.19/2.31 eV, respectively, in order of increasing energy. The difference between ADE and VDE reflects the degree of structural relaxation that occurs after electron detachment. A smaller difference suggests less relaxation, implying a more rigid geometry. Thus, isomer 1 is the most stable structure with minimal structural relaxation, making it energetically favourable in relaxed conditions. Isomer 2, the strongest electron binding and moderate flexibility, potentially making it favourable in dynamic or non-equilibrium situations.

In the anion case, isomer 5(b) shows a distortion in geometry compared to the neutral case of isomer 5(b). The bond lengths of Cu–Cu, Cu–In and Cu–Te are found to be 2.38 Å, 2.48 Å and 2.45 Å, respectively. The binding energy and HOMO–LUMO gap are calculated and found to be 0.927 eV and 1.023 eV, respectively.

### 3.2. Electronic properties of Cu_*m*−1_In_*m*_Te_2*m*−2_ neutral and anion clusters

**Fig. 6:**
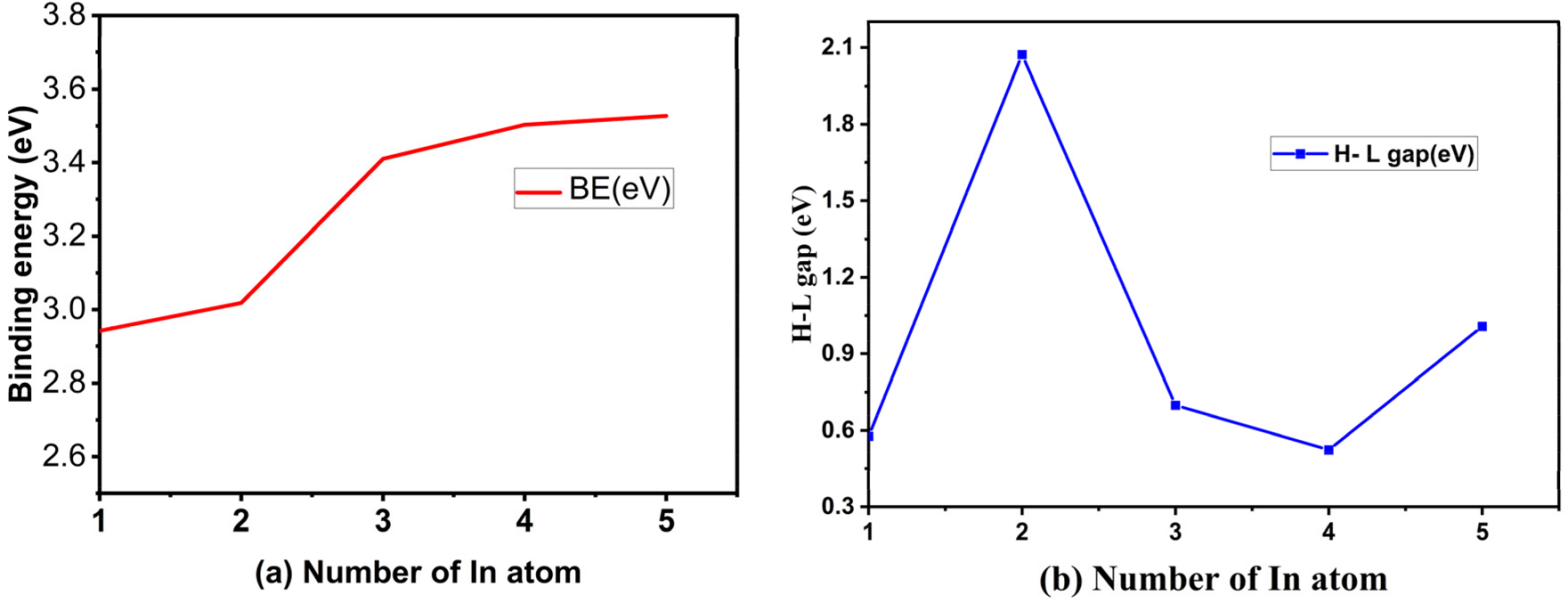
(a) Binding energy (eV) as a function of cluster size and (b) H–L gap energy (eV) as a function of cluster size of Cu_*m*−1_In_*m*_Te_2*m*−2_ for *m* = 2–5 neutral clusters.

The calculated result of BE (*m*), H–L gap, VDE, ADE and AIP value of the most stable structures of neutral Cu_*m*−1_In_*m*_Te_2*m*−2_ cluster are tabulated in Table 1. It is significant to calculate the atomic average binding energy to see the relative stabilities of the most stable Cu_*m*−1_In_*m*_Te_2*m*−2_ neutral clusters. Fig. 6 shows the size dependence of average binding energies BE (*m*) of Cu_*m*−1_In_*m*_Te_2*m*−2_ neutral clusters, which increase with the increasing number of clusters. It rapidly increases at *m* = 2 and slowly increases from *m* = 3 to 5, indicating that clusters 3, 4, and 5 have almost insignificant differences in energy and are considered as they approach the saturation level. This is supported by the small bond lengths contribute to the stability of various phases in Cu–Te compounds, allowing for dynamic stability under different conditions.^32^ BE (*m*) increases slightly as *m* increases, indicating stronger bonding with an increase in the number of atoms in the system. Larger systems (higher *m*) exhibit stronger bonding, likely due to increased coordination and interaction among the constituent atoms.

**Table 1:** Binding energy (eV), H–L gap (eV), vertical detachment energy, adiabatic detachment energy (eV) and adiabatic ionization potential (eV) for first low lying isomers of Cu_*m*−1_In_*m*_Te_2*m*−2_ (*m* = 2–5) neutral clusters

Cu_*m*−1_In_*m*_Te_2*m*−2_	BE	H–L gaps	VDE	ADE	AIP
CuInTe_2_	2.942	0.577	3.78	3.53	6.71
CuIn_2_Te_2_	3.018	2.072	3.83	3.69	6.16
Cu_2_In_3_Te_4_	3.410	0.699	3.92	3.76	5.60
Cu_3_In_4_Te_6_	3.503	0.524	4.58	3.68	4.89
Cu_4_In_5_Te_8_	3.527	1.007	4.34	3.60	4.96

The H–L gap decreases significantly as *m* increases, except for a slight increase in Cu_4_In_5_Te_8_. This indicates a narrowing of the energy gap between the highest occupied molecular orbital (HOMO) and the lowest unoccupied molecular orbital (LUMO), leading to increased metallic character. The decrease in H–L gap suggests increasing delocalization or metallic properties with system size, although Cu_4_In_5_Te_8_ indicates a slight restoration of semiconducting properties. While VDE increases, the ADE trend suggests that structural relaxation following electron removal is more significant in larger systems, thereby reducing the effective energy required for detachment.

We have discussed the electronic property of Cu_*m*−1_In_*m*_Te_2*m*−2_ by examining the energy gap between the HOMO and LUMO. Fig. 6(b) shows the size dependence of H–L gaps of Cu_*m*−1_In_*m*_Te_2*m*−2_ clusters. It is worthwhile to point out that we find an odd–even oscillating function with a most prominent peak at *m* = 3. As the size of the cluster increases, the HOMO–LUMO gap generally decreases due to reduced quantum confinement; however, in small clusters like those studied here, electronic properties often exhibit odd–even oscillatory behaviour driven by size-dependent quantum effects.^33^ At *m* = 3, the cluster size may achieve a balance between quantum confinement and electronic delocalisation, resulting in a particularly stable configuration with a larger energy gap. This stability could be attributed to a pseudo-closed-shell electronic structure, resembling the behaviour of “magic numbers” in nuclear physics or noble gas atoms, which are associated with greater stability and wider H–L gaps.^34^ Additionally, the symmetry and atomic arrangement at *m* = 3 likely favour optimal bonding and electron localization, further increasing the separation between the HOMO and LUMO energy levels. This symmetry-induced stabilization could lower the energy of the HOMO while raising the energy of the LUMO, thereby enlarging the gap. Furthermore, the specific interaction among copper, indium, and tellurium atoms at *m* = 3 may enhance orbital hybridization, stabilizing bonding molecular orbitals and destabilizing anti-bonding orbitals. The electronegativity of tellurium (Te) compared to copper (Cu) and indium (In) plays a significant role in influencing the electronic properties of semiconductor materials,^31^ particularly in the context of band gap variations. The electron-withdrawing effect of Te can lead to increased energy band gaps in compounds, which is crucial for their application in electronic and optoelectronic devices.^35^

The specific combination of factors, including electronic shell closure, symmetry-related stabilization, optimal hybridization, and quantum confinement effects, likely culminates at *m* = 3 to produce the largest H–L gap. Beyond this size, the increasing cluster size leads to reduced quantum confinement, and even-numbered clusters lose some of the electronic stabilization observed in odd-numbered clusters. This clearly shows as CuIn_2_Te_2_ owns the most enhanced stability. Hence, it is with an extraordinary enhanced chemical stability. Therefore, it can be considered as a novel candidate nano material.

**Fig. 7:**
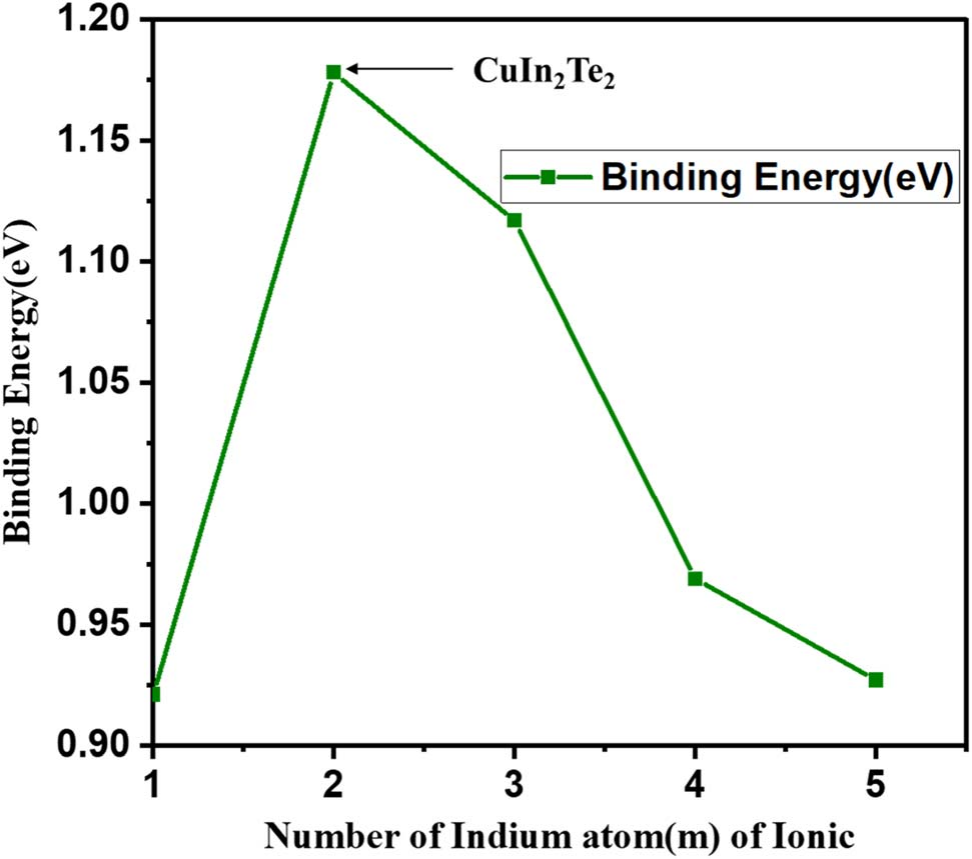
Binding energy (eV) for first isomers of anion (Cu_*m*−1_In_*m*_Te_2*m*−2_)^−^ for (*m* = 2–5) clusters as a function of cluster size *m*.

**Fig. 8:**
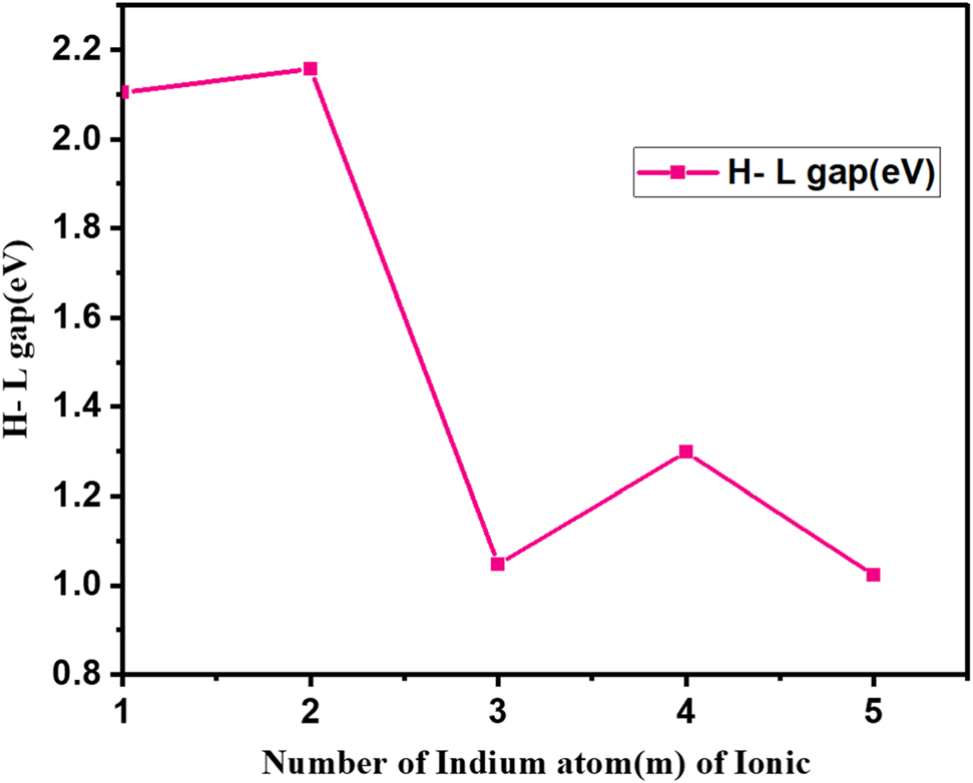
H–L gap (eV) for first isomers of anion (Cu_*m*−1_In_*m*_Te_2*m*−2_)^−^ for (*m* = 2–5) clusters as a function of cluster size *m*.

For the anion counterpart, the binding energy and H–L gap are tabulated in Table 2. Fig. 7 and 8 also show their plots as a function of the number of Cu_*m*−1_In_*m*_Te_2*m*−2_ anion clusters. The binding energy of (Cu_*m*−1_In_*m*_Te_2*m*−2_)^−^ clusters initially increases significantly from *m* = 1 to *m* = 2, peaking at *m* = 2. This indicates that the CuIn_2_Te_2_ cluster is the most stable configuration in the series, likely due to optimal electronic interactions and bonding among copper, indium, and tellurium atoms. The peak stability at *m* = 2 suggests a particularly favourable electronic structure or geometry, potentially driven by closed-shell configurations or enhanced orbital hybridisation, which minimises strain and maximises bonding efficiency.

**Table 2:** Bond length in (Å), binding energy (eV), and HOMO–LUMO gap (eV) for the first low-lying isomers of Cu_*m*−1_In_*m*_Te_2*m*−2_, (*m* = 2–5) anion clusters

Cu_*m*−1_In_*m*_Te_2*m*−2_	Cu–Cu	Cu–In	Cu–Te	In–Te	In–In	BE	H–L	VDE	ADE	AIP
(CuInTe_2_)^−^	**—**	2.47	2.51	2.61	**—**	**0.921**	2.104	3.78	3.53	6.71
(CuIn_2_Te_2_)^−^	—	2.67	2.38	3.08	2.97	1.178	2.157	3.83	3.69	6.16
(Cu_2_In_3_Te_4_)^−^	2.57	2.82	2.52	2.89	3.00	1.117	1.048	3.92	3.76	5.60
(Cu_3_In_4_Te_6_)^−^	2.65	2.45	2.50	2.75	3.14	0.969	1.298	4.58	3.68	4.89
(Cu_4_In_5_Te_8_)^−^	2.38	2.48	2.45	2.84	2.89	0.927	1.023	4.34	3.60	4.96

Beyond *m* = 2, the binding energy gradually declines as the cluster size increases (*m* = 3, 4, 5), indicating a decrease in stability. The electronic characteristics of relatively larger clusters deteriorate due to several interconnected causes, including reduced quantum confinement effects, less effective orbital overlap, and higher structural strain. As clusters become larger, these factors have a significant impact on their electrical structure and behaviour. The strain can also affect the confinement potential, resulting in a weaker confinement regime, which diminishes the overall electronic performance of the clusters.^36^ Additionally, the binding energy of nuclear systems exhibits minor fluctuations indicative of odd–even oscillations in stability. OES is observed in nuclear binding energies, where nuclei with odd mass numbers tend to have slightly higher binding energies compared to their even counterparts.^37^ This phenomenon is particularly evident in various fermionic systems, where odd–even staggering (OES) is a well-documented characteristic of binding energies. For instance, the energy at *m* = 3 is slightly higher than at *m* = 4, possibly due to differences in electronic configurations or geometric arrangements between odd and even-sized clusters. Together, these trends illustrate how cluster stability is influenced by size, geometry, and electronic structure. The decline in stability for larger clusters suggests that bonding is less efficient or that structural strain increases as size increases. Similar odd–even oscillations are noted in the stability of nanoclusters, where even-numbered clusters exhibit lower stability compared to odd-numbered ones. The underlying cause is linked to geometric and electronic structural differences that affect their reactivity and stability.^38^ This could be attributed to favourable electronic and geometric factors, such as hybridisation and closed-shell configurations. It is more imperative to note that the relatively shorter Cu–Te bond length in the (CuIn_2_Te_2_) cluster, as compared to neighbouring clusters, suggests stronger bonding interactions between Cu and Te atoms in this configuration. This enhanced bonding can lead to increased stability, which aligns with the observation that (CuIn_2_Te_2_) has the highest binding energy (BE = 1.178 eV) among the clusters. Stronger bonds may result from optimal hybridisation of orbitals between copper and tellurium atoms, promoting efficient overlap and charge transfer. As^39^ suggests, tellurium exhibits strong non-covalent interactions, comparable to hydrogen bonds, which can further stabilize the hybrid structures. This enhanced bonding reduces the bond length and could also contribute to a more compact and stable geometry, which is consistent with the peak in binding energy and the larger H–L gap 2.157 eV for (CuIn_2_Te_2_)^−^.

We have plotted the H–L gap of Cu_*m*−1_In_*m*_Te_2*m*−2_ anion as shown in Fig. 8. As shown in the plot, there is no significant difference in HOMO–LUMO gap between cluster *m* = 1 and 2, suggesting that the electronic structure and energy levels for clusters with *m* = 1 and 2 are relatively similar. This could imply that the addition of an extra unit (from *m* = 1 to *m* = 2) does not significantly alter the overall electronic environment. But there is a sharp peak at *m* = 2. Rapid decay from *m* = 2, 3 and oscillation afterwards is observed. Indicates that the cluster with *m* = 2 is exceptionally stable due to a pronounced increase in the H–L gap. This stability might be due to a closed-shell electronic configuration or enhanced symmetry, making the *m* = 2 cluster less reactive and more energetically favourable. Rapid decay from *m* = 2 to *m* = 3, implies that the stability of the clusters drops quickly as the size increases beyond *m* = 2. The addition of more units disrupts the favourable electronic configuration or symmetry that stabilizes the *m* = 2 cluster. The exceptional stability of the (Cu_*m*−1_In_*m*_Te_2*m*−2_)^−^ series at *m* = 2 can be attributed to favourable electronic and geometric configurations, which may stem from enhanced electron delocalization and optimal atomic coordination.

This stability is crucial for applications in nanotechnology and materials science, as it influences the structural integrity and performance of these compounds. In the context of alkanes, electron delocalization is linked to the stability of branched structures compared to linear isomers, indicating that similar principles may apply to the (Cu_*m*−1_In_*m*_Te_2*m*−2_)^−^ series.^40^ The sharp decline in the H–L gap from *m* = 2 to *m* = 3 suggests a shift to a less stable and more reactive regime. Beyond *m* = 3, the oscillatory gap behaviour reflects quantum size effects, where periodic stability changes occur as cluster size increases. These trends highlight the unique reactivity and stability properties of *m* = 2 clusters, making them ideal for stable applications (*e.g.*, optoelectronics), while larger clusters could be designed for tuneable electronic or reactive applications.

**Fig. 9:**
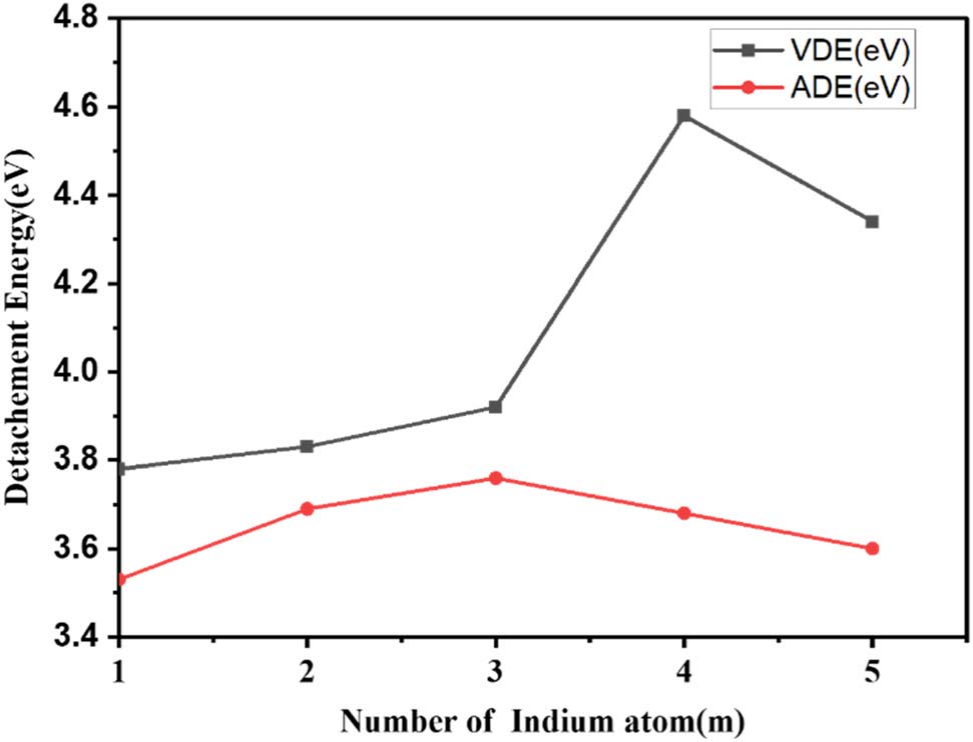
Vertical and adiabatic detachment energy of Cu_*m*−1_In_*m*_Te_2*m*−2_ for (*m* = 2–5) clusters as a function of cluster size *m*.

**Fig. 10:**
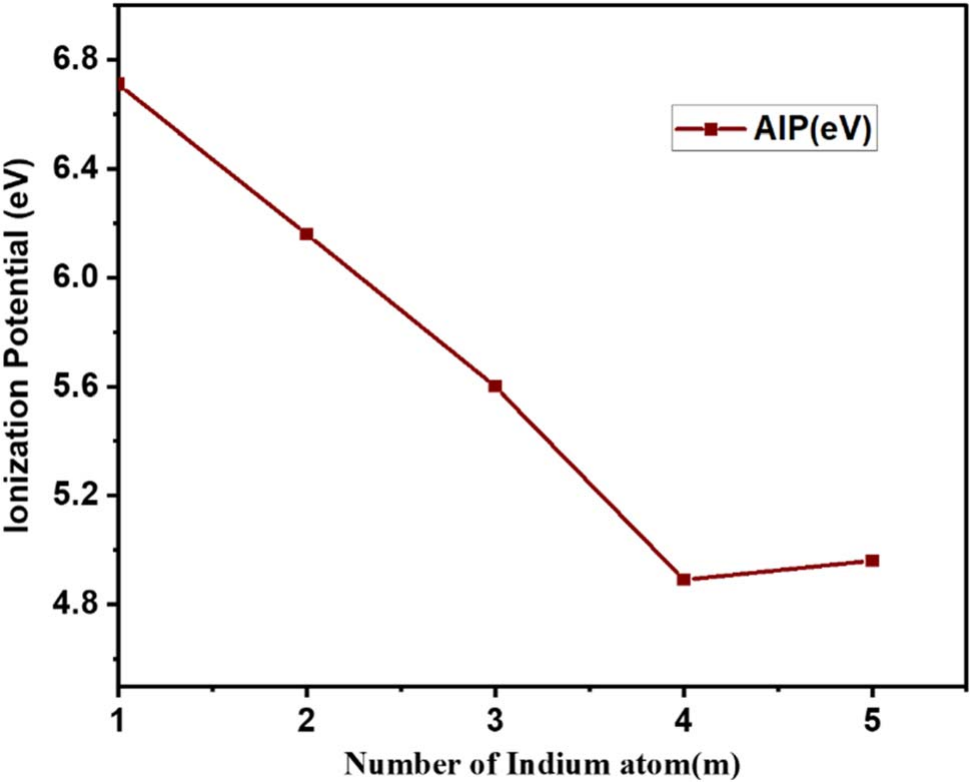
Adiabatic ionization potential of Cu_*m*−1_In_*m*_Te_2*m*−2_ for (*m* = 2–5) clusters as a function of cluster size *m*.

The ionization potentials and electron affinities of the clusters are additional sensitive characteristics that offer essential information about the electronic structure and chemical reactivity with other molecules. These characteristics are crucial for explaining how the electrical structure of clusters changes with size. As a result, we have determined the Cu_*m*−1_In_*m*_Te_2*m*−2_ (*m* = 2–5) clusters' Adiabatic Ionization Potential (AIP), Vertical Detachment Energy (VDE), and Adiabatic Detachment Energy (ADE). The detachment energies and ionisation potential as a function of cluster size are listed in Table 2 and plotted in Fig. 9 and 10, respectively. As shown in Fig. 9, it is obvious that a decreasing tendency can be seen when the number of Cu_*m*−1_In_*m*_Te_2*m*−2_ increases for AIP. It is known that the larger the AIP value is, the stronger the non-metallic characteristics will be, which can be associated with higher chemical inertness, and the larger the VDE is, the stronger the metallic characteristics. So the decrease in AIP indicates that the clusters' metallic characteristics become stronger. The successive addition of a metallic atom increases the metallic characteristics of the system. This leads to a decrease in AIP with increasing metallic atom.

From Fig. 10, we can see that the AIP decreases monotonically from *m* = 1 up to 5 and begins to oscillate afterwards. About the odd–even pattern of AIP, this is again a consequence of the electron pairing effect. In case of odd-*n* clusters with even valence electrons, the extra electron has to go into the next orbital, which costs energy, resulting in a lower value of AIP. Moreover, particularly large values for AIP are exhibited with *m* = 1. As cluster size increases, the delocalization of electrons becomes more pronounced, resulting in a lower energy requirement for electron removal and a decrease in ionization potential. This phenomenon marks a transition from molecular-like behaviour in smaller clusters to bulk-like properties in larger clusters, where ionization potentials are generally lower than those of isolated atoms or smaller clusters. The following sections elaborate on this transition. This is also supported by ref. 41, in larger clusters, electrons are more delocalized, which reduces the energy needed for ionization. The delocalization is linked to the geometric structure of clusters, transitioning from distinct molecular arrangements to more uniform bulk-like structures.^42^ One contributing factor is the higher surface-to-volume ratio in smaller clusters, where a larger fraction of atoms resides at the surface, causing electrons to be more strongly localized and resulting in higher ionization energies. In contrast, as the cluster size increases, the proportion of surface atoms decreases, reducing surface-specific effects and further lowering the ionization potential. Additionally, larger clusters exhibit higher polarizability, which allows them to stabilize the electron cloud more effectively, further decreasing the energy required for ionization. Together, these factors underscore the evolution of electronic and structural properties as clusters grow in size. This interestingly shows a strong non-metallic favour is reflected compared to neighbouring clusters.

### 3.3. Partial charge density analysis clusters

The study focuses on the electronic properties of clusters, particularly the density of states (DOS), to understand the size effects of the clusters. The partial density of states (PDOS) is examined based on the contributions from different orbital components (s, p, d), as well as the highest occupied molecular orbital (HOMO) and lowest unoccupied molecular orbital (LUMO) is surfaces for the Cu_*m*−1_In_*m*_Te_2*m*−2_ clusters. A high DOS at a specific energy level indicates many available states for occupation, while zero DOS means no states at that energy. The total DOS can be broken down into the contributions from s, p, and d orbitals. Due to the small size of the clusters, quantum confinement effects lead to discrete energy levels, unlike bulk systems which have continuous energy bands. The DOS is presented as a series of spikes, reflecting these discrete energy levels.

#### 3.3.1. Partial charge density analysis Cu_*m*−1_In_*m*_Te_2*m*−2_ neutral clusters

The calculated partial charge density for occupied and unoccupied orbitals of (CuIn_2_Te_2_) and (CuIn_2_Te_2_)^−^ are tabulated in Table 3. As we can see in the table, for HOMO levels of both the neutral and anion clusters, the molecular orbitals have contributions mainly from In and Te atoms. The In contributions are from the majority s and minority p orbitals, and the Te atom contribution is from the p orbital only. The HOMO−1 is basically localised on the p orbital of the Te atom, s and p orbitals of In, and dominantly from the d orbital of the Cu atom. There is also a minor localisation from the p orbital of the In atom and a major contribution from the p orbital of the tellurium atom of the anion CuIn_2_Te_2_ clusters. For neutral and anion cases, the LUMO level major contributions are from p-orbitals of both In and Te atoms with a minor contribution from the Cu atom. There is no contribution from s orbital of all atoms for neutral clusters. The main contributor to LUMO+1 of the neutral is the p-orbital of Te and In atoms. For the anion part, the main contributor for LUMO+1 is the P orbital of In and Te, with a minor contribution from the P orbital of the Cu atom. We clearly see that the electronic state at the nearest of the Fermi level comes mainly from p orbitals of Te atoms and d orbitals of Cu atoms for both neutral and anion ones.

**Table 3:** s, p and d partial charges within Cu, In and Te atoms calculated for some occupied and unoccupied orbitals in (CuIn_2_Te_2_) (right) and (CuIn_2_Te_2_)^−^ (left) clusters

Orbital	Atom	s	p	d	Orbital	Atom	s	p	d
HOMO−1	Cu	0.000	0.007	0.102	HOMO–1	Cu	0.000	0.013	0.274
	In	0.061	0.052	0.001		In	0.000	0.030	0.003
	In	0.061	0.052	0.001		In	0.000	0.030	0.003
	Te	0.001	0.104	0.001		Te	0.002	0.140	0.000
	Te	0.001	0.104	0.001		Te	0.002	0.140	0.000
HOMO	Cu	0.000	0.006	0.007	HOMO	Cu	0.000	0.006	0.007
	In	0.070	0.037	0.002		In	0.065	0.035	0.001
	In	0.070	0.037	0.002		In	0.065	0.035	0.001
	Te	0.000	0.155	0.001		Te	0.000	0.155	0.000
	Te	0.000	0.155	0.001		Te	0.000	0.155	0.000
LUMO	Cu	0.010	0.005	0.037	LUMO	Cu	0.002	0.001	0.022
	In	0.001	0.116	0.003		In	0.000	0.138	0.002
	In	0.001	0.116	0.003		In	0.000	0.138	0.002
	Te	0.002	0.035	0.006		Te	0.000	0.046	0.003
	Te	0.000	0.035	0.006		Te	0.000	0.046	0.003
LUMO+1	Cu	0.003	0.000	0.029	LUMO+1	Cu	0.000	0.027	0.006
	In	0.000	0.012	0.001		In	0.001	0.126	0.002
	In	0.000	0.012	0.001		In	0.001	0.126	0.002
	Te	0.000	0.067	0.005		Te	0.000	0.039	0.005
	Te	0.000	0.067	0.005		Te	0.000	0.039	0.000

**Fig. 11:**
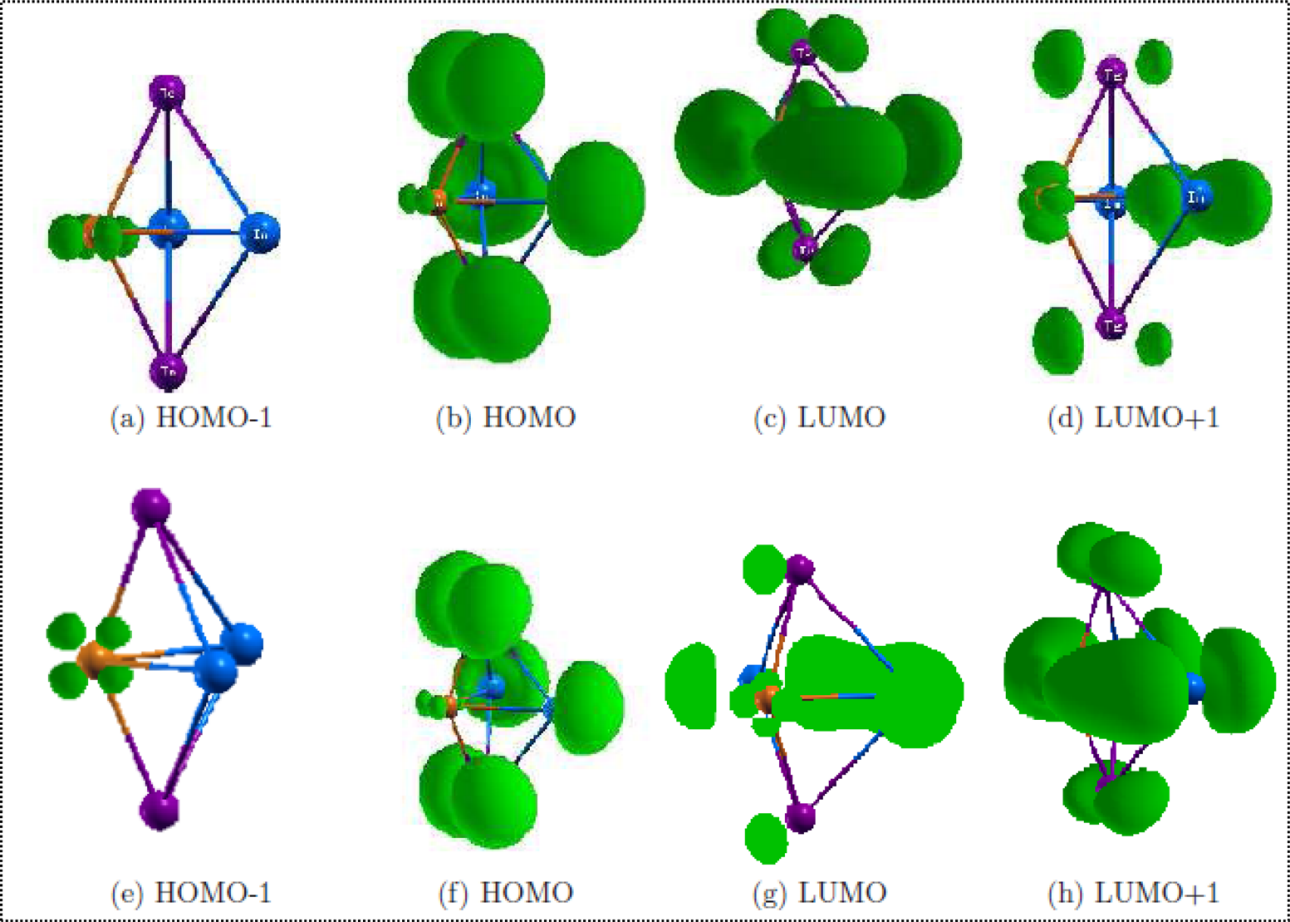
Partial charge density plots of the HOMO−1, HOMO, LUMO and LUMO+1 orbitals of (CuIn_2_Te_2_) (above) and (CuIn_2_Te_2_)^−^ (below). Subsurface plots are at 1/5th of the maximum value.

The analysis underscores the central roles of Te (p orbitals) and Cu (d orbitals) in determining the electronic states near the Fermi level for both neutral and anion clusters, while In contributes through its s and p orbitals to both occupied and unoccupied states (Fig. 11). The differences between neutral and anion clusters, particularly the minor involvement of Cu in unoccupied states, highlight the subtle impact of anion charge on orbital contributions. Overall, the Te and Cu atoms emerge as the primary contributors to the electronic properties near the Fermi level, with Te dominating both valence and conduction states and Cu having a more localized influence on deeper occupied and higher unoccupied states. This is strongly in line with,^43^ the strong hybridization between Te and Cu enhances the metallic character of the electronic states near the Fermi level. Cu's d orbitals primarily affect deeper occupied and higher unoccupied states, showing minor involvement in unoccupied states for anion clusters.

**Fig. 12:**
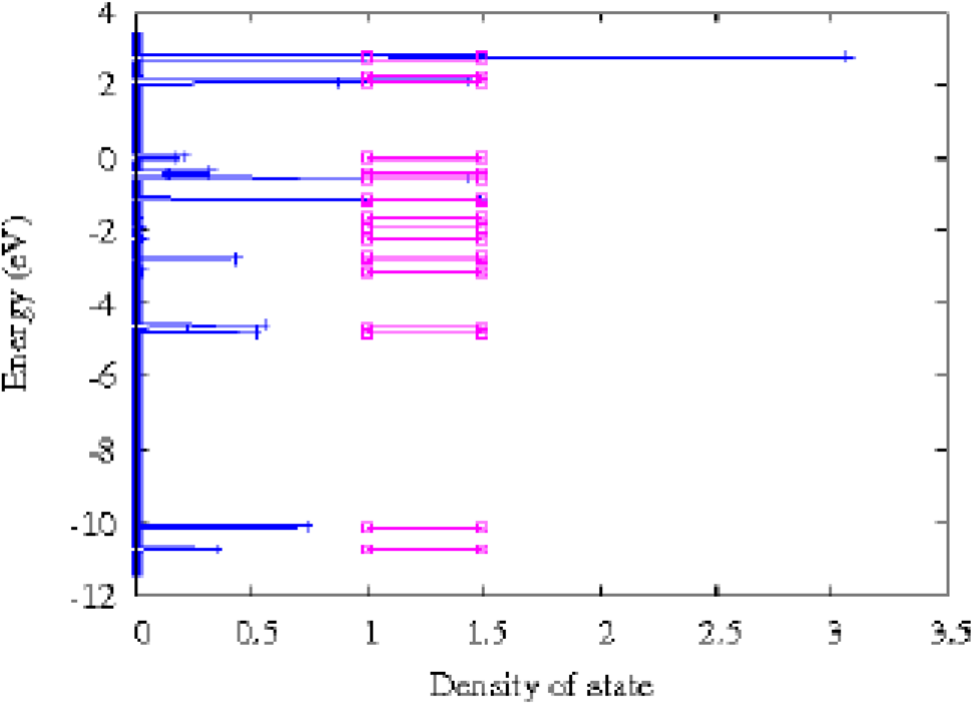
LDOS and energy levels of CuIn_2_Te_2_ clusters. The Fermi level is shifted to the zero of the energy axis. The discrete spectra are broadened by a Gaussian of width 0.01 eV.

We know that the Fermi energy is the average of energy of HOMO and energy of HOMO–LUMO gap. A close inspection of the LDOS plot (Fig. 12) combined charge density analysis presented in Table 3 reveals that near the Fermi level, the LDOS levels higher than the Fermi levels have dominant contribution from Te and Cu atoms of neutral and anion CuIn_2_Te_2_ clusters whereas the levels with LDOS lower than the Fermi level have dominant contribution from In and Te atoms. From the LDOS plot in Fig. 12 shows that, due to increment in indium atom and tellurium, there is shift of energy bands to the higher level for example, the HOMO energy level of the CuInTe_2_ is −5.158 eV with reference to the Fermi level after adding indium atoms the HOMO energy level becomes −4.886 eV. This clearly indicates a shift in energy bands.

## 4. Summary and conclusion

In this work structural and electronic properties of Cu_*m*−1_In_*m*_Te_2*m*−2_ neutral and anion clusters are studied using QUANTUM ESPRESSO/PWSCF package based on the principle of density functional theory (DFT), which in turn depends on pseudo-potential with a plane wave basis sets. For the optimization of energy and geometry conjugate gradient algorithm is used to find the localized minimum energy geometry. The main calculated results are summarized as follows. First of all, many initial configurations have been optimized to obtain the lowest energy structures for Cu_*m*−1_In_*m*_Te_2*m*−2_ (*m* = 2–5) clusters using conjugate gradient algorithm. Our optimized geometries indicate that, copper atom is centre seeking embedded inside sharing the highest coordination bond with neighbours rather than extending outside. In the study of the structural property of a certain cluster, measuring the bond length between two atoms is one of the structural properties of a cluster but whatever we measure the average bond length; it will never exceed the bond length of the bulk. The atomic average binding energy, H–L gap, and the second-ordered difference of energy as a function of cluster size of Cu_*m*−1_In_*m*_Te_2*m*−2_ neutral and anion clusters are studied. An important prerequisite for the chemical stability and less reactivity is a large H–L gap. In this perspective among the minimum energy structures of Cu_*m*−1_In_*m*_Te_2*m*−2_ (*m* = 2–5) clusters, CuIn_2_Te_2_/(CuIn_2_Te_2_)^−^ possess large H–L energy gap. This indicates that these two clusters, earn high chemical stability indicating that they are inert to participate in any chemical reaction with their environment. We have observed that for most of the optimized geometry of Cu_*m*−1_In_*m*_Te_2*m*−2_ neutral and anion clusters' H–L gap is in the range of 0.524–2.464 eV. This numerical value enables us to conclude that, both clusters have novel electronic and structural property than their adjacent clusters. Hence, they have valuable and ultimate contribution for semiconductor designing, photovoltaic solar cell and storage devices. As electronic structure of both clusters at nano level can be controlled, the H–L gap of the clusters for example can be adjusted by manipulating their size and shape. Dealing of the molecular orbital like HOMO and LUMO levels of a certain cluster can show us the location of the conduction molecular orbital of that given cluster. In addition to the above mentioned parameters of relative stability, ionization potential and electron affinities were also parameters which are used to measure relative stability of both clusters. The vertical and adiabatic detachment energies as well as adiabatic ionization potential as a function of cluster size were plotted. Both adiabatic detachment energy (ADEs) and vertical detachment energy (VDEs) increase for cluster size increase. (Cu_3_In_4_Te_6_)^−^ has the highest VDE implies that the anionic form is relatively close in energy to its corresponding neutral form in the optimized geometry of the anion. This reflects a high electron affinity for the neutral species. It also suggests a system with strong electronic stability, high electron affinity, and low propensity for electron loss. This is valuable for applications in materials science, molecular electronics, and catalysis. As the ADE value shows, the (Cu_*m*−1_In_*m*_Te_2*m*−2_)^−^ clusters exhibit high stability across the series, with a slight maximum in stability at *m* = 3. This indicates that the electronic and structural configurations are well-balanced in these clusters, with potentially interesting properties around *m* = 3 due to enhanced stabilization. The trend in ionization potential reflects the interplay between electronic delocalization and structural compactness in these clusters. Smaller clusters (*m* = 1, 2) exhibit high IP values due to stronger electron localization, while larger clusters (*m* = 3, 4, 5) have lower IP values due to increased delocalization. The slight stabilization in IP at *m* = 5 suggests the emergence of a stable electronic and structural configuration in larger clusters. The objectives of this work have been addressed and this could be a good foundation for further work in the area of chalcopyrite of I–III–VI_2_. As a recommendation, studying the electronic structure of copper indium di telluride (CIT) nano particle with doping at an appreciable size and composition is the future work in the area of thin film solar cells. The analysis presented in this study can also be extended qualitatively to other semiconductor systems and computational results may stimulate further study on self-assemble clusters leading to further applications in photovoltaic thin film solar cells and optoelectronics.

## Author contributions

Kidane G., writing the original draft, conceptualization, investigation, editing and detail discussion, Dessie A., Tesfay G., Ashenafi B., Ayalew M., were participated in reviewing and editing of the manuscript. Hagos Weldeghebriel Zeweldi was participated in supervising, technical matter, reviewing, resources, conceptualization, and editing of the manuscript. All authors agree with the final form of a manuscript.

## Conflicts of interest

There are no conflicts to declare.

## Data Availability

All data supporting the findings of this study are available within the article. Additional details or computational files can be provided by the corresponding author upon reasonable request.
